# Numerical Simulation and Experimental Verification of Multi-Probe Cryoablation

**DOI:** 10.3390/mi16121321

**Published:** 2025-11-25

**Authors:** Jian Zhang, Bei Tong, Changmao Ni, Guoting Fu, Binglei Pan, Li Huang

**Affiliations:** Wuhan Neuracom Technology Development Co., Ltd., Wuhan 430073, China; zhangjian@neuracom.com.cn (J.Z.); tongbei@neuracom.com.cn (B.T.); nichangmao@neuracom.com.cn (C.N.); fuguoting@neuracom.com.cn (G.F.); panbinglei@neuracom.com.cn (B.P.)

**Keywords:** cryoablation, numerical simulation, multi-probe layout, ex vivo validation

## Abstract

This paper addresses the challenges of ice ball shape prediction and layout optimization in multi-probe cryoablation treatment through a comprehensive study integrating both simulation and experimental approaches. A three-dimensional numerical model of multi-probe cryoablation, coupled with phase change heat transfer, was developed using the Pennes bioheat equation. The model’s accuracy in predicting core physical phenomena, such as phase change processes and multi-probe thermal field superposition, was initially validated. The simulated and experimentally measured temperature profiles, along with the macroscopic ice ball shapes, were observed to be in excellent agreement (with an average error of 3.75% in the major and minor axes of the ice ball). Additionally, it was determined that a nine-probe layout with 1 cm probe spacing was optimal for generating a uniform low-temperature field. However, histological analysis of porcine liver tissue revealed inconsistencies between the model’s predicted damage boundaries and the actual observed diffuse biological damage transition zone, indicating the limitations of steady-state models relying solely on fixed critical damage threshold temperatures for accurately predicting the cell death region. This study not only provides a rigorously validated thermal-physical prediction tool for preoperative planning but also underscores the importance of incorporating time-temperature thermal dose effects into future models to bridge the gap from physical simulation to biological damage prediction, thus laying a crucial foundation for the development of precise cryoablation technologies.

## 1. Introduction

Hepatocellular carcinoma (HCC) ranks among the most prevalent malignancies worldwide, characterized by persistently elevated incidence and mortality rates [1]. For patients with early-stage liver cancer who are not candidates for surgical resection, local ablative therapy has become a cornerstone of clinical intervention [2]. Within this therapeutic arsenal, cryoablation represents a prominent physical ablation technique. It utilizes cyclical processes of extreme freezing and active thawing to induce irreversible coagulative necrosis within the tumor tissue. This modality offers distinct advantages, including its minimally invasive nature, cost effectiveness, and ability to stimulate a systemic anti-tumor immune response [3].

Notwithstanding the demonstrated efficacy of cryoablation, a significant limitation persists: the ice ball generated by a single cryoprobe is often constrained in size, rendering it insufficient for effectively covering tumors, which are typically irregularly shaped and of substantial volume [4,5]. Consequently, clinical practice necessitates a multi-probe synergistic strategy. The pivotal challenge in achieving personalized and precise cryotherapy lies in the accurate pre-procedural prediction of the final ice ball morphology and the associated temperature field distribution resulting from the interaction of multiple probes. This predictive task is considerably complicated by the pronounced heterogeneity of tissue thermophysical properties, which can introduce deviations in the anticipated ice ball volume, thereby leading to potential miscalibrations of the ablation zone [6,7,8]. Furthermore, the inability of clinicians to accurately discern the progression and configuration of the evolving ice ball based solely on the relative positioning of the cryoprobes may undermine both the therapeutic efficacy and safety of the procedure. Therefore, the development of a high-fidelity computational model for cryoablation is imperative, serving as a critical tool to support clinicians in optimizing preoperative planning.

Numerical simulation technology presents a viable approach to addressing the aforementioned challenges. By establishing multi-physics coupling models based on the bioheat equation, this technique facilitates the intuitive visualization of the ablation process under various surgical parameters, thereby providing a crucial evidence base for optimizing therapeutic protocols [9,10,11]. However, the current body of scholarly inquiry remains predominantly concentrated on simulations involving single or dual probes. The complex configurations necessitating a greater number of probes (e.g., ≥5) remain largely underexplored [12]. A more significant limitation is that the predictive accuracy of many existing models has not been substantiated through systematic and rigorous experimental validation. There is a particular dearth of a structured, phased validation process progressing from homogeneous tissue-mimicking phantoms to ex vivo biological tissues. This deficit inevitably raises concerns about the reliability of their predictive outcomes and, thus, their potential for clinical translation [13,14,15,16].

The present study establishes a numerical model for cryoablation that incorporates the latent heat of phase change, using the finite element software COMSOL Multiphysics 6.2 and adopting a multi-physics coupling approach. This model is employed to elucidate the dynamic influences of probe configuration and quantity on the evolving morphology of the ice ball and the distribution of critical isotherms. A systematic comparative analysis between numerical simulations and experimental validations was performed for various configurations, including single-probe, five-probe, and nine-probe arrays. This investigation specifically focuses on examining the impact of probe number and spatial arrangement on the ultimate ablation outcome. The findings of this research are intended to provide a theoretical foundation for enhancing the precision of multi-probe cryoablation procedures and minimizing collateral damage, thereby advancing the optimization of personalized treatment strategies.

## 2. Materials and Methods

This study employs a phased, progressive validation strategy to rigorously evaluate the reliability of the multi-probe cryoablation numerical model. The initial phase involves a comparative analysis between simulation outcomes and experiments conducted on structurally and thermophysically homogeneous potatoes. This preliminary stage is designed to isolate and validate the core physical processes embedded within the model—namely, phase-change heat transfer and the interaction of multi-probe thermal fields—while deliberately excluding the confounding complexities inherent in perfused living tissues. Subsequently, the model’s predictive reliability is assessed in a context more closely resembling biological tissue through direct comparison with experimental data from ex vivo porcine liver tissue. All numerical simulations presented in this section are configured and benchmarked against the experimental parameters established in Phases 1 and 2. Furthermore, an anatomically realistic geometry model was constructed based on clinical liver CT data, representing the ultimate clinical application target of this research. A detailed simulation analysis of this model, incorporating physiological parameters, represents a key focus for future investigation. This section will provide a comprehensive description of the universal numerical methodology employed across all simulations, as well as the specific experimental setups for both validation phases.

### 2.1. Numerical Modeling

The present numerical study was conducted using the COMSOL Multiphysics 6.2 software environment to solve the transient bioheat transfer equation. All computations were performed on a high-performance workstation equipped with an 11th Gen Intel^®^ Core™ i7-11800H processor (@ 2.30 GHz) and 32 GB of RAM, utilizing parallel processing capabilities to enhance computational efficiency.

The thermal distribution within the tissue during the cryoablation process was characterized using the classical Pennes bioheat model [17]. This governing equation was selected for its parsimonious formulation, which incorporates the essential effects of metabolic heat generation and blood perfusion while retaining computational tractability. Its widespread adoption in the literature further affirms its utility for such analyses. The mathematical expression of the model is given by Equation (1) [18]:

(1) $$\rho c\frac{\partial T}{\partial t}=k\nabla^{2}T+w_{b}\rho_{b}c_{b}(T_{b}-T)+Q_{m}$$

where *ρ* denotes tissue density (kg/m^3^), c represents tissue specific heat capacity (J/(kg·°C)), T signifies local tissue temperature (°C), and k defines tissue thermal conductivity (W/(m·°C)). w_b_ represents the blood perfusion rate (s^−1^); *ρ_b_* defines blood density (kg/m^3^); c_b_ represents blood specific heat capacity (J/(kg·°C)); and Tb defines the temperature of arterial blood (°C). Finally, Q_m_ represents the volumetric metabolic heat generation rate within the tissue (W/m^3^).

Given that the cryoablation process involves the liquid-solid phase transition of biological tissues and the associated release and absorption of latent heat, the thermophysical properties of liver tissue are altered during the phase change. This study employs the effective heat capacity method to simulate the phase transition process, whereby the material’s thermophysical parameters are modified by defining a transient temperature-dependent transformation fraction. The temperature-sensitive parameters in liver tissue include specific heat capacity, thermal conductivity, and density. The computational formulae for these three parameters are given in Equations (2)–(4) as follows [19]:

(2) $$k=\left\{\begin{array}{ccc} k_{u} & T_{mu}<T & \\ \frac{k_{u}+k_{f}}{2} & T_{ml}⩽T⩽T_{mu} & \\ k_{f} & T<T_{ml} & \end{array}\right.$$

(3) $$\begin{array}{c} c=\left\{\begin{array}{ccc} c_{u} & T_{mu}<T & \\ \frac{c_{u}+c_{f}}{2}+\frac{Q_{lf}}{\left(T_{mu}-T_{ml}\right)} & T_{ml}⩽T⩽T_{mu} & \\ c_{f} & T<T_{ml} & \end{array}\right. \end{array}$$

(4) $$\begin{array}{c} \rho =\left\{\begin{array}{ccc} \rho_{u} & T_{mu}<T & \\ \frac{\rho_{u}+\rho_{f}}{2} & T_{ml}⩽T⩽T_{mu} & \\ \rho_{f} & T<T_{ml} & \end{array}\right. \end{array}\;$$

For model simplification, both normal and tumor tissues are assumed to be isotropic and homogeneous, and are considered to be initially in a liquid phase. Throughout the entire cryoablation process, the tissue may exist in one of three states: liquid phase, solid phase, or a solid–liquid mixed phase. When the temperature exceeds the upper phase transition threshold T_mu_, the tissue remains in the liquid phase; when the temperature lies between the lower phase transition threshold T_ml_ and the upper threshold T_mu_, it transitions into a solid–liquid mixed phase; and when the temperature further decreases below T_ml_, the tissue is considered fully frozen and in the solid phase [20,21].

The thermal response and damage development in biological tissue are characterized by a temperature-threshold framework that synthesizes energy conservation, heat conduction dynamics, and the kinetics of tissue damage. The governing equations are as follows [19]:

(5) $${\left(\rho C_{p}\right)}_{eff}\frac{\partial T}{\partial t}+{\left(\rho C_{p}\right)}_{eff}u\cdot \nabla T+\nabla \cdot q=Q+Q_{bio}$$

(6) $$q=-k_{eff}\nabla T\;$$

(7) $${\left(\rho C_{p}\right)}_{eff}=\theta_{d}\rho_{d}C_{p,d}+\left(1-\theta_{d}\right)\rho C_{p}$$

(8) $$k_{eff}=\theta_{d}k_{d}+\left(1-\theta_{d}\right)k\;$$

(9) $$Q=\rho L_{d,c}\frac{\partial \theta_{d}}{\partial t}\left(T<T_{d,c}\right)$$

(10) $$\theta_{d}=\min\left(\alpha ,1\right)$$

(11) $$\frac{\partial \alpha }{\partial t}=\frac{1}{t_{d,c}}\left(T<T_{d,c}\right)$$

where the damage temperature threshold (T_d,c_) is set to −20 °C, the damage time (t_d,c_) to 60 s, the necrosis temperature (T_n,c_) to −50 °C, and the enthalpy change (L_d,c_) to 250 kJ/kg.

**Figure 1:**
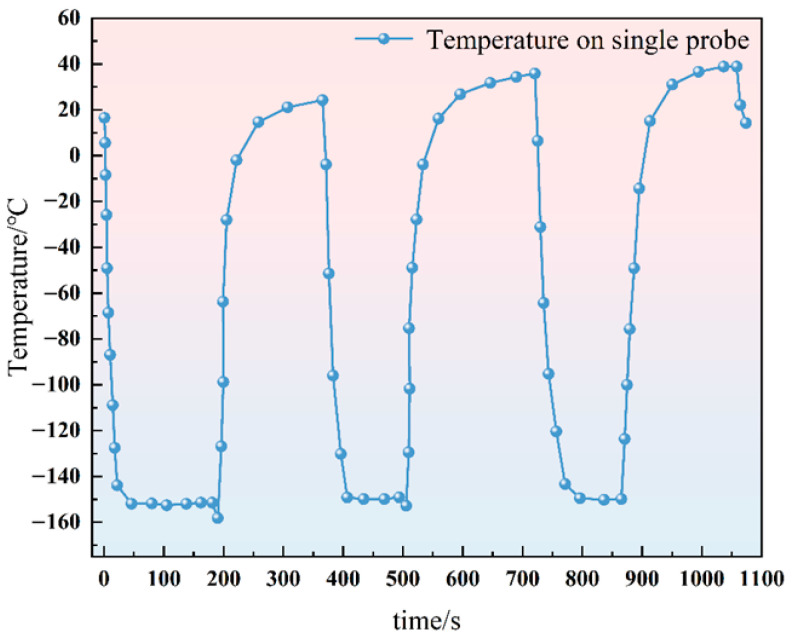
Single probe temperature load curve.

The external boundaries of the computational domain and the non-cooling portions of the cryoprobes were defined as thermally insulated. The initial condition for the tissue was set to the normal human core temperature of T_0_ = 37 °C, while the active cooling segment of the cryoprobe was subjected to a prescribed temperature boundary condition derived from experimental measurements, as illustrated in Figure 1. The relevant thermophysical parameters utilized in the present numerical model are summarized in Table 1. The material parameters of probe are listed in Table 2.

**Table 1:** Material Parameters for Tissue Models [22].

Parameter	Description	Unit	Magnitude	Note
k	Conductivity of tissue	W/(m·℃)	0.5	Liquid Phase
			2	Solid Phase
c	Specific heat of tissue	J/(kg·℃)	3600	Liquid Phase
			1800	Solid Phase
ρ	Density of tissue	kg/m^3^	1000	Liquid Phase
			998	Solid Phase
c_b_	Specific heat of blood	J/(kg·℃)	3600	-
*w_b_*	Blood perfusion rate	s^−1^	0.0005	-
Q_m_	Metabolic heat generation	W/m^3^	4200	-
Q_lf_	Latent heat of phase transition	MJ/m^3^	250	-
T_mu_	Upper limit of phase transition	℃	−1	-
T_mf_	Lower limit of phase transition	℃	−8	-

**Table 2:** Material Parameters for probe.

Material	Conductivity	Density	Specific Heat
Nitinol	21.9 [W/(m*K)]	4506 [kg/m^3^]	522 [J/(kg*K)]
PU	0.18 [W/(m*K)]	930 [kg/m^3^]	1900 [J/(kg*K)]

### 2.2. Numerical Method Verification

**Figure 2:**
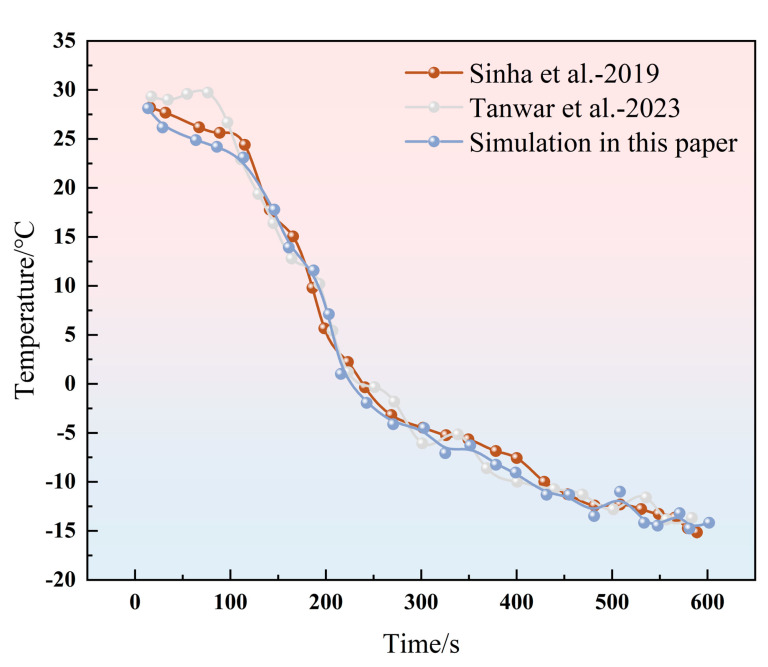
Numerical method verification, comparing with Sonam and Sinha [17,23].

To validate the accuracy of the cryoablation numerical model in characterizing heat transfer and tissue injury, a single-probe cryoablation simulation was conducted. The simulation was first benchmarked by reproducing the in vitro experiments of Sinha et al. and the numerical simulations of Tanwar et al. [17]. Adopting the experimental parameters from Sinha et al. [23], the temporal temperature profile along the cryoprobe axis at 10 mm from the tip was extracted and comparatively plotted in Figure 2 below.

**Figure 3:**
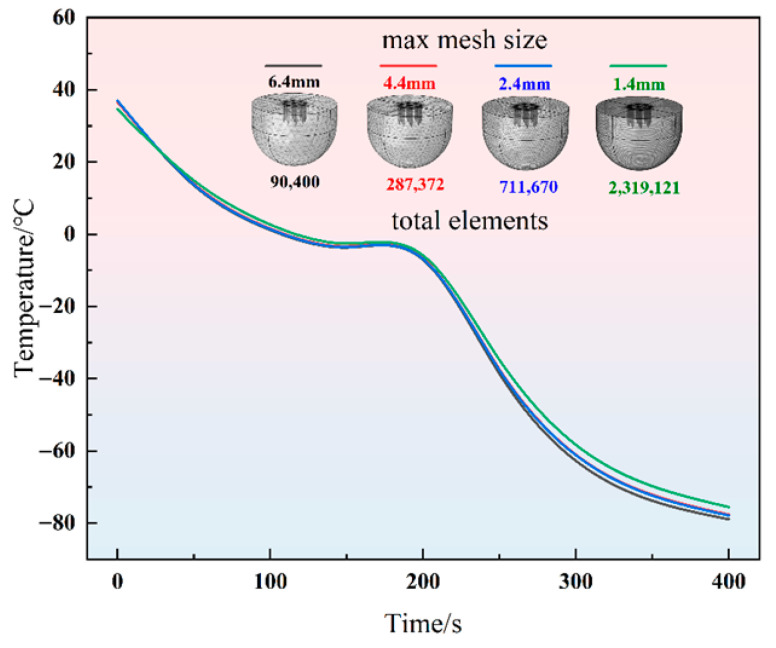
Temperature curve of FE model with four maximum mesh sizes.

Following the validation of the numerical methodology, a grid independence study was conducted for the heat transfer model. Four distinct numerical models with maximum grid sizes of 6.4 mm, 4.4 mm, 2.4 mm, and 1.4 mm were used to perform transient bioheat transfer simulations. Figure 3 illustrates the temperature profiles at a distance of 5 mm from the probe tip for these four different grid resolutions. As the mesh is progressively refined, the temperature curves exhibit a converging trend; however, the substantial increase in the total number of elements leads to a surge in computational time. Overall, a maximum grid size of 2.4 mm ensures the convergence of the numerical results while keeping computational costs within an acceptable range. Consequently, this grid resolution was adopted for all subsequent computations.

### 2.3. Experimental Setup

This section describes the experimental validation of the numerical model, which was structured into two phases. Initial method verification and equipment checks were performed qualitatively using potato phantoms, while a subsequent quantitative validation and direct model comparison were conducted using ex vivo porcine liver tissue to better simulate biological conditions.

**Figure 4:**
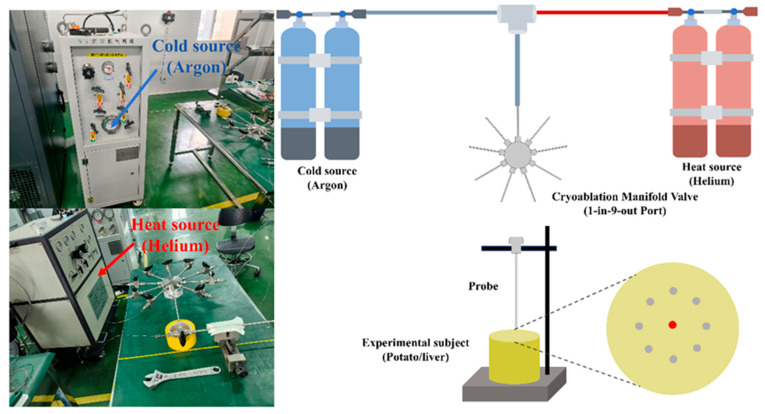
Experimental setup and schematic diagram.

The experimental system comprised a thermal source unit providing cooling via high-pressure argon and active heating via high-pressure helium. We deployed nine identical cryoprobes and recorded temperatures using a 4-channel USB DAQ with T-type thermocouples at 1 Hz. A custom flange fixture guaranteed precise, repeatable positioning for single, five, and nine-probe layouts, with all probes inserted to a depth of 2 cm. The experimental layout and its schematic are shown in Figure 4.

Initial validation and qualitative assessment of ice ball formation were performed on fresh potato tubers, chosen for their homogeneous high water content, to verify the phase change modeling approach. A more rigorous, biologically relevant validation was then conducted on ex vivo porcine liver. The liver specimens, obtained within 2 h post-slaughter, were refrigerated and tested within 4 h to ensure freshness. Experiments utilized lobes greater than 5 cm thick to mitigate thermal boundary effects during cryoablation.

The experimental procedure was conducted as follows. The specimen (potato or liver tissue) was placed on a platform maintained at room temperature (25 °C). A cryoprobe, mounted onto the fixture, was inserted into the specimen to a depth of 2 cm. Four thermocouple measurement points were established: at the main probe, 5 mm from the main probe, at an auxiliary probe, and at a distal reference location. A 15 min freeze–thaw cycle was subsequently initiated, with temperature data recorded throughout the entire process.

Upon conclusion of the experiment, the potato specimens were sectioned transversely and longitudinally. Their maximum transverse and longitudinal dimensions were measured using calipers. The porcine liver samples were fixed in formalin, sectioned, and subjected to Hematoxylin and Eosin (H&E) staining and TUNEL staining to assess cellular apoptosis and necrosis. Each experimental configuration was repeated in triplicate to ensure reproducibility.

It is noteworthy that all porcine livers used in this study were sourced as by-products of the food industry, with no animals sacrificed specifically for this experimental work.

## 3. Results and Discussion

### 3.1. Thermophysical Validation: Prediction of Ice Ball and Temperature Field

**Figure 5:**
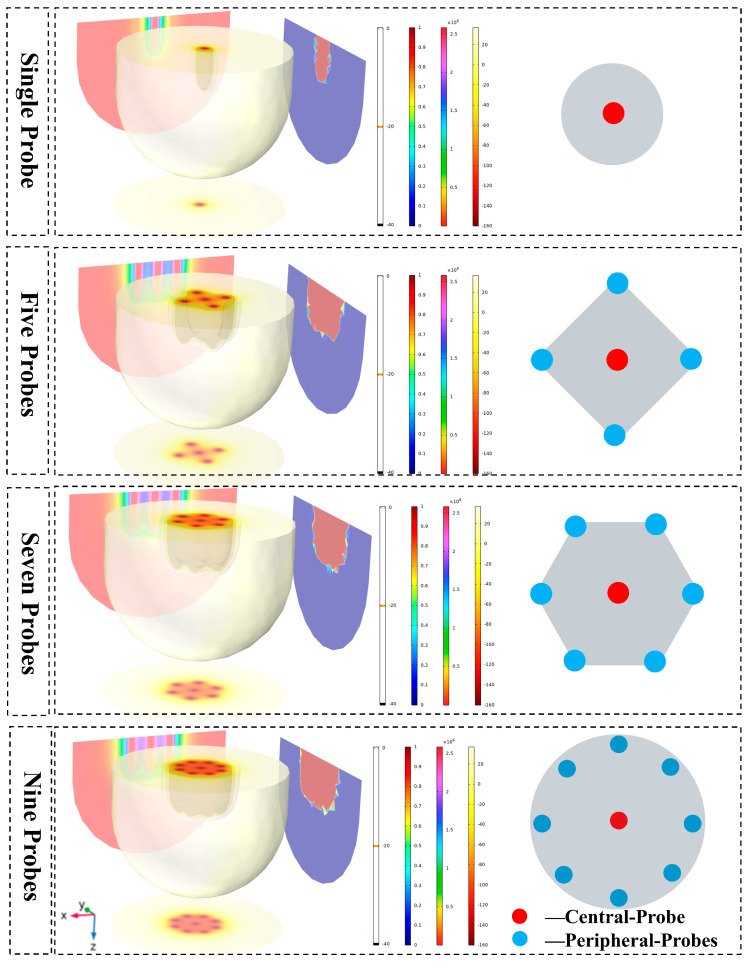
Under the same freezing time (t = 900 s), the simulation result show the temperature field, the morphology of the ice ball (0 °C isotherm), and the damage fraction for the four layouts.

Based on the thermophysical parameters and boundary conditions derived from the potato experiments, numerical models were established for single, five, seven, and nine-probe configurations. Figure 5 presents the simulated contour plots of the temperature field, the ice ball morphology (represented by the 0 °C isotherm), and the damage fraction for the four layouts at an identical freezing time (t = 900 s).

As illustrated in Figure 5, the single-probe configuration generated a symmetrical, ellipsoidal ice ball with maximum longitudinal and transverse dimensions of 2.5 cm and 1.2 cm, respectively. The five-probe annular arrangement produced a larger, composite ice ball; however, pronounced warm zones, where temperatures exceeded the critical threshold for irreversible cellular damage (−40 °C), were evident within the interstitial regions of the array. The seven-probe layout effectively eliminated the aforementioned warm zones and significantly expanded the coverage of the cryoablative area, although the resulting ice ball still exhibited an irregular, multi-lobed (“plum-blossom”) morphology. The nine-probe matrix configuration demonstrated the most comprehensive performance, generating the largest, most regular, and nearly cylindrical composite ice ball. Within this configuration, the vast majority of the interior volume attained temperatures below −40 °C, indicating the most uniform and complete ablation coverage achievable among the layouts tested.

**Figure 6:**
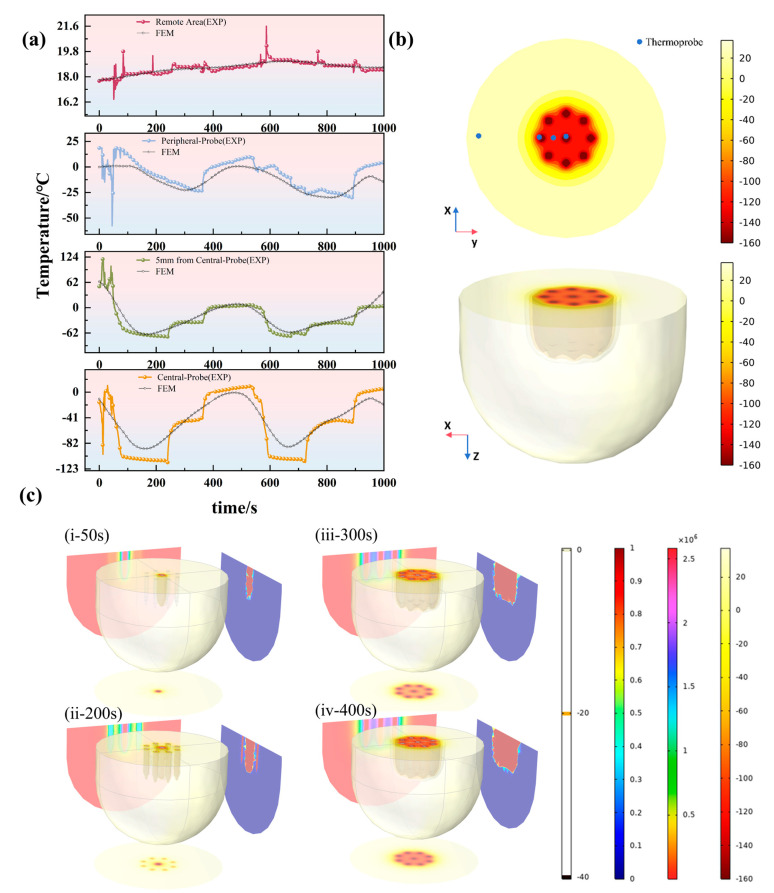
(**a**) temperature curve of EXP and FEM; (**b**) temperature field of FEM; (**c**) contour plots at four critical time points (t = 50 s, 200 s, 300 s, and 400 s).

To further investigate the cryodynamics of the optimal configuration, we monitored the temporal evolution of the temperature field throughout the entire procedure for the nine-probe layout. Figure 6 displays the simulated contour plots at four critical time points (t = 50 s, 200 s, 300 s, and 400 s). The evolutionary process clearly reveals that during the initial freezing phase (t = 50 s), the ice balls around individual probes grew independently. As the process continued (t = 200 s), the advancing ice fronts from adjacent probes began to contact and coalesce, forming a continuous frozen region. By the intermediate stage (t = 300 s), the ice ball continued to expand outward while the temperature within the interior region decreased more uniformly. The lethal zone, enclosed by the −40 °C isotherm, steadily enlarged until the end of the freezing cycle, ultimately resulting in the formation of a large, homogeneous composite ice ball capable of completely covering the target area. This progression visually validates the physical mechanism of “independent growth → fusion → collective expansion” inherent to multi-probe configurations.

**Figure 7:**
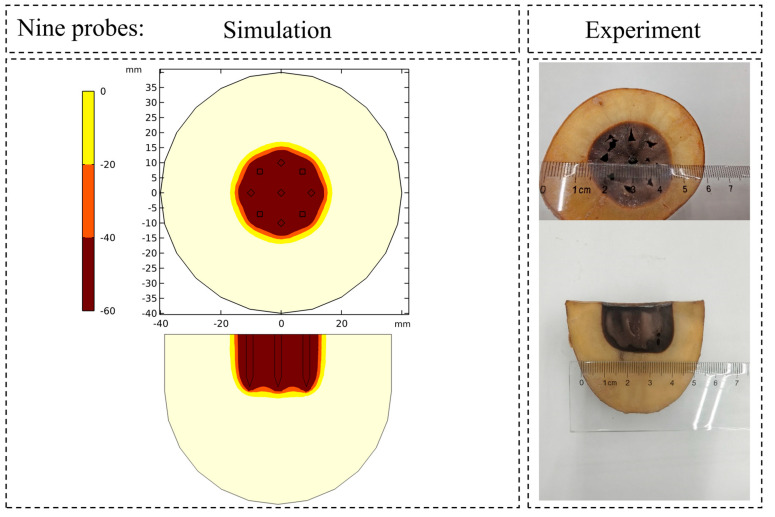
Comparison of the ice ball contour simulated by the nine-probes layout with the experimentally observed damage zone.

**Figure 8:**
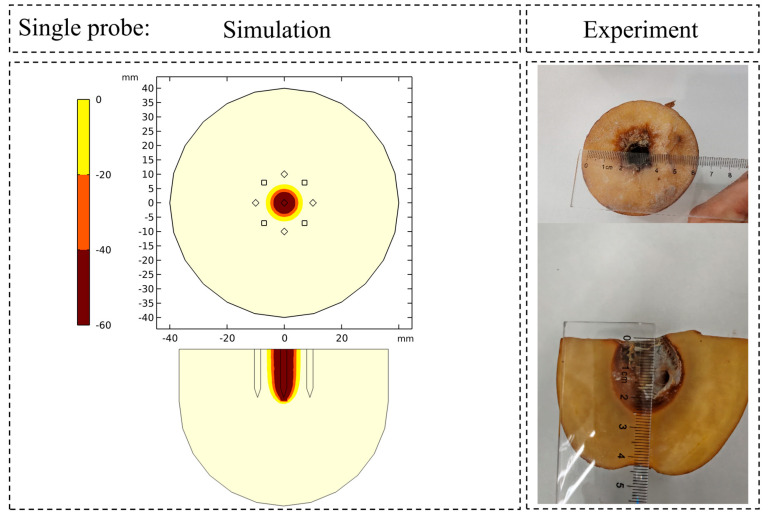
Comparison of the ice ball contour simulated by the single probe layout with the experimentally observed damage zone.

The accuracy of the potato-based numerical model was validated through direct comparison of the simulated 0 °C isotherm against macroscopic cross-sectional views of the experimentally induced damaged zones in potato specimens. As evidenced in Figure 7 and Figure 8 for both the single-probe and nine-probe configurations, the simulated ice ball contours demonstrate remarkable agreement with the experimentally observed damaged regions in both shape and dimensions. Quantitative analysis revealed a mean discrepancy of 3.75% in the primary dimensions (long and short axes) between the simulation and experimental results. These findings collectively validate the high precision of the numerical model in capturing core physical phenomena, including multi-probe interactions, phase change heat transfer, and the resulting morphology of the ice ball.

**Figure 9:**
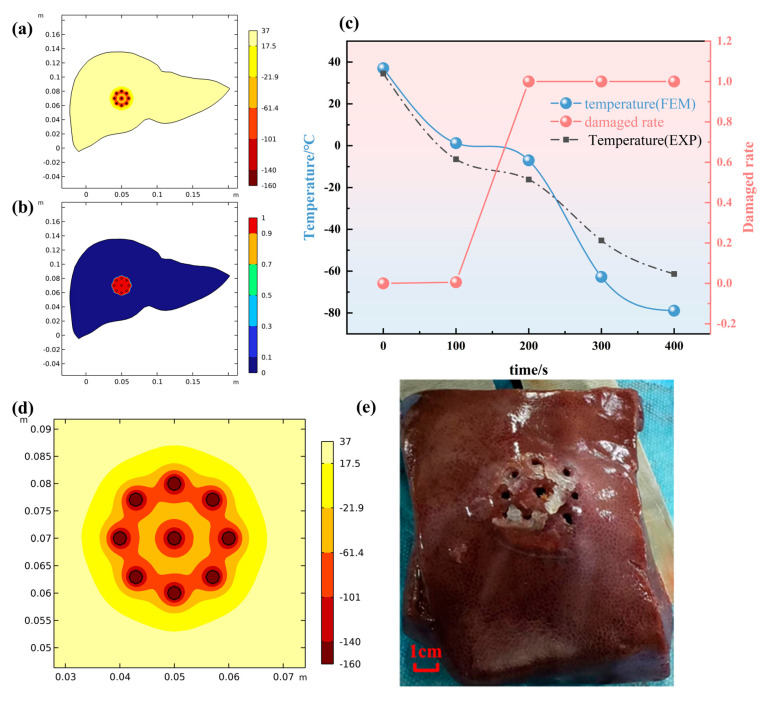
(**a**) temperature field of simulation in liver; (**b**) damaged rate of simulation in liver; (**c**) temperature curve and damaged rate of EXP and FEM; (**d**) magnified view of the temperature field isosurface; (**e**) photo of liver after experiment (without probes).

A further comparative analysis between the ex vivo porcine liver experiments and the corresponding simulation results is presented in Figure 9. Figure 9a,b depict the simulated isothermal surfaces and damage fraction contours for the nine-probe configuration, while Figure 9c shows the temporal temperature profiles and the corresponding tissue damage fractions from both experiment and simulation. It can be observed that the predicted damage boundary from the simulation agrees well with the experimentally observed pale frozen zone in both overall morphology and dimensions, with a mean dimensional error of approximately 7.5%. Furthermore, the simulated temperature histories demonstrate close alignment with the experimental measurements across all monitored locations. The thermal damage fraction curves also exhibit consistent evolutionary trends, collectively indicating that the model reliably captures the essential biothermal phenomena during the multi-probe cryoablation process, including temperature distribution and the attainment of the irreversible damage threshold.

### 3.2. Biological Damage Assessment

While the numerical model demonstrates proficiency in predicting the temperature field and macroscopic ice ball morphology, the ultimate efficacy of cryoablation is determined by its capacity to induce irreversible cellular damage. To evaluate the model’s predictive performance regarding tissue ablation injury, ex vivo porcine liver samples were subjected to Hematoxylin and Eosin (H&E) and TUNEL staining analyses. The resultant histological findings were subsequently benchmarked against the simulated damage regions to assess predictive accuracy.

**Figure 10:**
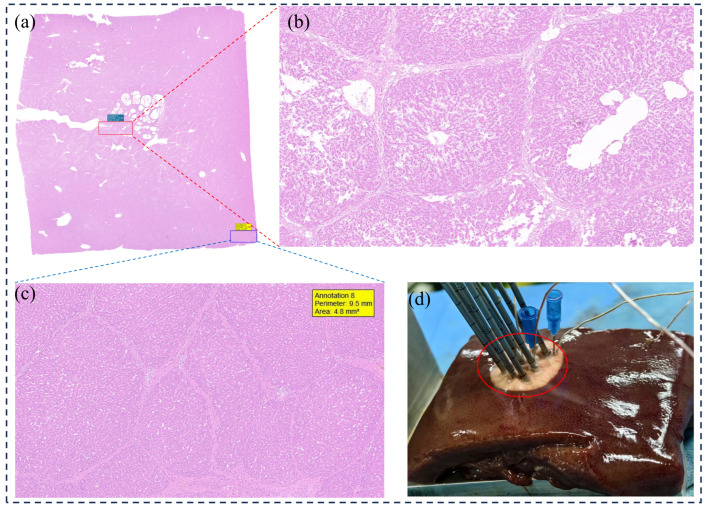
(**a**–**c**) H&E staining results; (**d**) damaged area during experiment.

**Figure 11:**
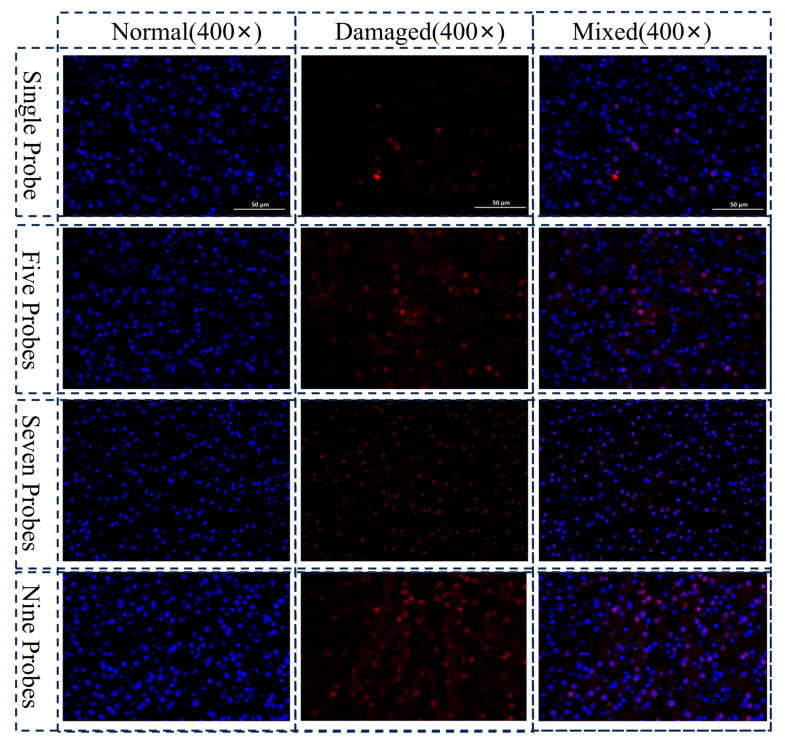
TUNEL staining results of different layouts.

Figure 10 and Figure 11 present the representative H&E and TUNEL staining results, respectively. Within the macroscopic ice ball boundary, the H&E staining reveals extensive zones of cellular structural disruption, exemplified by characteristic features of coagulative necrosis including nuclear pyknosis, karyorrhexis, and enhanced eosinophilic cytoplasm. Complementing these observations, TUNEL staining demonstrates a pronounced positive signal in the corresponding regions, indicating widespread DNA fragmentation consistent with the advanced stages of both apoptotic and necrotic cell death pathways.

However, neither the H&E nor TUNEL staining revealed a sharply delineated boundary of tissue injury. In contrast to the simulation, which predicted a distinct demarcation between completely necrotic and apparently healthy tissue, histological examination identified a transitional zone where extensively necrotic tissue intermingled with structurally intact yet biochemically compromised (TUNEL-positive) cells. This spatial heterogeneity results in a histologically diffuse injury boundary that diverges from the clear demarcation produced by the model. Furthermore, the conventional −40 °C threshold for irreversible cellular damage, implemented in the simulation, failed to manifest as a distinct histological boundary in the experimental results.

The presence of TUNEL-positive signals confirms the induction of apoptosis. Being an active and time-consuming process, apoptotic progression likely lags behind the rapid physical events of freezing. Consequently, the tissue damage observed at the experiment’s conclusion represents a superposition of immediate physical effects and subsequent biochemical cascades, resulting in a spatially diffuse injury boundary. Furthermore, liver tissue comprises hepatocytes, vascular endothelial cells, cholangiocytes, and other constituents, each exhibiting distinct cryosensitivity. The model’s homogeneous tissue assumption oversimplifies this inherent microstructural heterogeneity, thereby contributing to the discrepancy in predicting the global damage boundary. Additionally, the absence of blood perfusion in ex vivo tissues alters their metabolic activity and response mechanisms compared to in vivo conditions, potentially influencing the ultimate manifestation and progression of cryo-induced injury.

The discrepancy between the simulated sharp damage boundary and the histologically observed diffuse transition zone can be further attributed to two key biological and physical factors not fully captured by the steady-state model. First, cell death is not an instantaneous function of temperature alone but is governed by a time-temperature thermal dose effect. The cumulative exposure time to sub-lethal temperatures influences the extent of damage, meaning that regions briefly reaching −40 °C may not undergo complete necrosis, whereas regions sustained at slightly higher temperatures for a prolonged period might. Second, the assumption of biological homogeneity is a simplification. Real liver tissue comprises a heterogeneous mix of cell types—hepatocytes, vascular endothelial cells, and cholangiocytes—each exhibiting distinct inherent cryosensitivity or ‘fitness’ to withstand thermal stress. This biological variation inevitably results in a graded, rather than a binary, response to freezing, contributing to the blurring of the definitive injury boundary observed in histology.

### 3.3. Limitations and Future Works

While this study has established a highly reliable numerical model for predicting the thermophysical fields during cryoablation through a multi-stage experimental validation process and has provided insights into the biological damage response, it is not without its limitations, which also chart a course for future research.

• A primary limitation arises from the use of ex vivo porcine liver tissue. While its thermal properties approximate human liver, the critical absence of hemodynamics is not accounted for. In vivo, the “heat sink” effect of major vessels would profoundly alter the ice ball’s shape, consequently resulting in incomplete ablation. This discrepancy poses a foremost clinical challenge that lies beyond the predictive scope of the present model.
• Secondly, the computational model simplifies the liver as a homogeneous, isotropic material, thereby omitting the complex microstructural heterogeneity—including bile ducts, vasculature, and lobular architecture—found in real tissue. This omission of microscale thermophysical variations is a plausible explanation for the mismatch between the simulated lesion boundary and the histologically defined injury zone.
• Thirdly, this study observed a diffuse injury boundary but lacked a quantitative, automated image-analysis protocol to define this transitional zone precisely. The reliance on qualitative and semi-quantitative comparisons thus constrained further analysis of the spatial principles governing its extent.

Furthermore, it is crucial to acknowledge that a degree of inherent predictive uncertainty will always persist, even with more sophisticated models. This uncertainty stems from the inter-patient variability in hepatic vasculature and the precise, patient-specific values of thermophysical parameters, which are difficult to ascertain a priori and may change dynamically during the procedure. Consequently, while computational models like ours are invaluable for preoperative planning and probe layout optimization, their predictions must not be interpreted as absolute boundaries in a clinical setting. To ensure complete tumor eradication and account for this uncertainty, the application of a safety margin beyond the simulated lethal zone is an indispensable clinical imperative.

Based on the aforementioned limitations, we propose the following future research directions: to conduct in vivo animal experiments and, building upon this foundation, to enhance the current model into a porous media model coupled with computational fluid dynamics. By incorporating the influence of hepatic vasculature and blood perfusion, this initiative will significantly improve the model’s predictive accuracy and practical utility in real clinical scenarios.

## 4. Conclusions

This study developed and validated a three-dimensional numerical model for preoperative planning of multi-probe hepatic cryoablation. Through a phased validation protocol, progressing from tissue-mimicking phantoms to ex vivo liver tissue, the model demonstrated high fidelity in predicting the spatiotemporal evolution of temperature fields and iceball morphology. It reliably simulated the phase-change processes for both single-probe and complex multi-probe configurations, with the nine-probe matrix layout identified as optimal for generating a large, uniform low-temperature field. Histological analysis revealed a critical insight: while the model predicted a distinct ablation boundary, the actual tissue exhibited a diffuse transitional zone. This discrepancy underscores the limitation of relying solely on a critical temperature isotherm for predicting cellular demise and reinforces the importance of the time-temperature thermal dose effect, alongside the synergistic mechanisms of apoptosis and necrosis. The established model serves as an effective tool for preoperatively assessing the ablation zone and optimizing probe placement. For clinical application, incorporating a safety margin beyond the simulated iceball boundary is recommended. Future research will focus on integrating hemodynamic perfusion and biophysical damage kinetics models, thereby providing a more robust theoretical foundation for the development of a precision cryoablation system.

## Data Availability

The original contributions presented in this study are included in the article. Further inquiries can be directed to the corresponding author.
